# Providing predictive models for quality parameters of groundwater resources in arid areas of central Iran: A case study of kashan plain

**DOI:** 10.1016/j.heliyon.2024.e31493

**Published:** 2024-05-17

**Authors:** Aysan Morovvati Zarajabad, Mahdi Hadi, Ramin Nabizadeh Nodehi, Mahsa Moradi, Mohammad Rezvani Ghalhari, Abbas Zeraatkar, Amir Hossein Mahvi

**Affiliations:** a Department of Environmental Health Engineering, School of Public Health, Tehran University of Medical Sciences, Tehran, Iran; b Center for Water Quality Research (CWQR), Institute for Environmental Research (IER), Tehran University of Medical Sciences, Tehran, Iran; c Center for Monitoring Water and Wastewater Sanitation, Kashan Water and Wastewater Company, Kashan, Iran

**Keywords:** Chemical parameters, Groundwater, Modeling, Water quality

## Abstract

Groundwater pollution can occur due to both anthropogenic and natural causes, leading to a decline in water quality and posing a threat to human health and the environment. The pollution of ground water resources with chemical pollutants is often considered. To manage water resources sustainably, ensuring their quality and quantity is crucial. Yet, testing groundwater can be expensive and time-consuming. So, using modeling to predict the chemical parameters of groundwater resources is considered to be an efficient and economical method. In this study, we examined three models to predict groundwater quality in dry regions by using R programming language. The random forest (RF) outperformed the other models in developing predictive models for water quality. Also, the multiple linear regression (MLR) model demonstrated strong performance, particularly in predicting total hardness (TH) in Aran Va Bidgol groundwater resources. The decision tree (DT) model did well but had lower performance than the RF model in predicting quality parameters. This approach can be efficacious in the field of effective management and protection of groundwater resources and enables the assessment of risks related to water resources.

## List of abbreviations

**Abbreviation terms**
Ca: Calcium
Cl: Chloride
DT: Decision Tree
EC: Electrical Conductivity
F: Fluoride
Fe: Iron
HCO_3_: Bicarbonates
K: potassium
Mg: Magnesium
MLR: Multiple Linear Regression
Mn: Manganese
Na: Sodium
NH_3_: Ammonia
NO_2_: Nitrite
NO_3_: Nitrate
pH: Potential of Hydrogen
PO_4_: Phosphate
R^2^: Coefficient of determination
RF: Random Forest
RMSE: Root Mean Squared Error
SO_4_: Sulphate
TA: Total Alkalinity
TDS: Total Dissolved Solids
TH: Total Hardness

## 1 Introduction

Access to an adequate quantity of fresh water, with suitable quality, is essential for achieving sustainable development. Groundwater resources serve as essential sources of freshwater, especially in arid and semi-arid regions characterized by minimal annual precipitation and frequent drought conditions [1]. They are known as very sensitive and endangered resources due to the negative effects of various human activities [2]. As a result of consuming polluted water, health issues are increasing globally; mostly leading to a rise in the rates of morbidity and mortality, especially among children [3]. Therefore, water quality assessment has a fundamental role in environmental management and scientific-based decision-making for the protection and rational exploitation of water resources.

In many fields, conducting large-scale sampling is impractical due to its high cost and limited resources. Consequently, there is a need for monitoring approaches that are more cost-effective and expedited [4]. Simulation models, with their predictive capabilities, often serve as the sole feasible means for analyzing input data and facilitating management decision-making [5].

Predictive modeling and preventive strategies have become popular in empowering policymakers to effectively manage groundwater through informed decisions and recommendations [6]. Meanwhile, multiple linear regression (MLR) models the relationship between two or more independent variables and a dependent variable by fitting a linear equation to the observed data [7]. Decision tree (DT) models are widely used in data mining, as they can model predictors with assumptions of dependence between them [8]. Random forest (RF) is a widely used machine learning algorithm that has been extensively applied in water resources studies in recent years. It enables the creation of predictive models through group regressions [9].

regarding this issue, various studies have been done to provide predictive models of groundwater quality parameters [3,[10], [11], [12], [13], [14], [15], [16], [17]]. Application of MLR models to predict the fluoride content of groundwater in Maharashtra, India showed good performance of the regression analysis. Also, the ground water resources of this area were suitable for drinking. the values of pH, HCO_3_^−^ and F^−^ were higher than the optimal level only in a small number of samples [18]. Using the DT algorithm to predict the water quality index of the Klang River in Malaysia showed the efficiency and cost-effectiveness of the proposed model, because it had a significant ability to simplify, analyze and classify raw data to reduce its complexity and non-linearity [8]. Investigating the performance of random forest regression to create predictive models of nitrate pollution in aquifers located in southern Spain showed the ability of this algorithm to build accurate models with strong predictive capabilities. Also, the developed model showed the probability of nitrate concentrations exceeding 50 mg/liter in this aquifer [19]. Chemical analysis of 75 water samples from Miandoab plain aquifer using random forest and fuzzy model found good groundwater quality. The RF was reliable for investigating groundwater vulnerability and management due to high accuracy, learning non-linear relationships, and identifying important variables [9]. The modeling of water quality parameters using MLR showed satisfactory results in estimating total dissolved solids(TDS) and electrical conductivity(EC), and it was also proposed as one of the basic tools in the field of water resources management and design [20]. The RF model was a valuable tool in evaluating water quality parameters and ensuring safe drinking water consumption. It performed well in predicting the TDS [21]. The goal of this study is to develop predictive models for total hardness (TH), total dissolved solids (TDS), and total alkalinity (TA) in water quality based on other quality parameters. Also, to compare the performance of these models with each other. According to the wide application of regression models and machine learning in recent studies, The MLR models, DT and RF algorithms were developed to predict the values of quality parameters of groundwater resources. Modeling of groundwater quality data during 9 years (2013–2021) was done by developing codes and arguments in R programming language.

## 2 Materials and methods

### 2.1 Study area

**Fig. 1:**
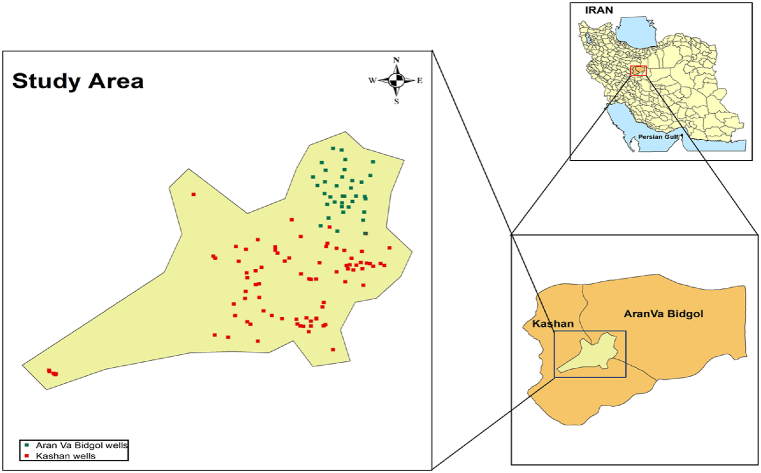
location of wells in the study area.

Kashan is one of the largest cities in Isfahan province, central region of Iran and located in 51.58°E 33.98°N. It has a hot, dry and desert climate. Aran Va Bidgol, another city in central region of Iran, is located 51. 28°E 34. 3°N and has an elevation of 840–985 m above the sea level. The average annual rainfall in Kashan and Aran Va Bidgol is 138.1 mm and 120 mm, respectively. As a result of the decrease in groundwater levels caused by water extraction and drought in these regions, numerous reports have been published on the occurrence of land subsidence in recent years. Also, the water crisis in both regions has led to increased concern about surface and groundwater resources, both in terms of quantity and quality. Fig. 1 depicts the well locations within the study area.

### 2.2 Data collection

The water and wastewater company of Kashan provided data on the quality parameters of groundwater resources. This data included quality test results from 84 wells in Kashan (including 6 in Barzok) and 36 wells in Aran Va Bidgol over 9 years (2013–2021). The water samples were collected and tested using standard methods at the company's laboratory in Kashan. In this study, a separate dataset was created for the wells of each city.

### 2.3 Data wrangling

It entailed cleaning, organizing, removing duplicates, correcting errors, ensuring quality, validating data, and filling missing values to ensure its quality and reliability in Excel 2019.Finally, the data were formatted in a way that was suitable for analysis in R software version 4.2.1.

### 2.4 Modeling of quality parameters of groundwater

In order to provide predictive models for the quality parameters of the groundwater resources of Kashan and Aran Va Bidgol, the data sets of the quality parameters of each city called in to the environment R software version 4.2.1. Correlation between quality parameters was investigated using corrplot software package using cor () function with Pearson method. Considering the importance of TH, TDS and TA in water quality studies, in this study predictive models were created for these three parameters based on other qualitative parameters.

By examining the correlation between the investigated parameters for each dependent variable, the desired independent variables were identified for developing models. Then, by installing the tidymodels package and using the set. seed (), initial_split (), training () and testing () functions, the data were randomly divided into two training and testing groups. So that 75 % of the data was placed in the training group for learning and fitting the model and 25 % of the data was placed in the testing group to evaluate the performance of the created model. Set seed () function was used to randomly allocate data in training and testing groups and make them comparable in determining the performance of models in predicting each desired parameter. In this way, the same data is used for each response parameter in the development of all three predictive models. To develop predictive models, three approaches: MLR, DT and RF were used.

#### 2.4.1 Multiple linear regression (MLR)

MLR analyzes the relationship between multiple independent variables and a continuous dependent variable white a simple linear regression. The MLR model was fitted using the linear_reg () function and the training group data separately for three response variables: TH, TDS and TA for both cities. The performance of the models using the predict () function by coefficient of determination (R^2^) was measured for both training and testing groups. Also, the rmse_vec () function was used to calculate the Root Mean Squared Error (RMSE) for the predicted values.

#### 2.4.2 Decision tree (DT)

In the context of regression the DT model, one of the machine learning algorithms, builds models in the form of a tree structure. As the algorithm progresses, an associated decision tree is incrementally developed. At first, by using the decision_tree () function, decision tree arguments were optimized, the engine was adjusted, and the regression mode was selected. Then, using grid_regular (), vfold_cv () and workflow () functions, the decision tree model was fitted for three water quality parameters. With the final fitting of the developed model, the performance of the models was measured using the predict () function for two training and testing groups with R^2^. Also, the rmse_vec () function was used to calculate the RMSE for the predicted values.

#### 2.4.3 Random forest (RF)

The RF approach, another machine learning algorithms, is an ensemble method that combines multiple decision trees to make predictions. It was applied for modeling of TH, TDS and TA using the rand_forest () function by choosing the ranger engine and the regression mode. The model was fitted on training data with recipe (), workflow (), tune_grid () and last_fit () functions. By selecting the best model using the select_best (metric = "rmse") function, the values of the hyperparameters mtry () and min_n () were obtained. Then, by applying the obtained values, the final fitting of the model was done.

### 2.5 Model evaluation

A common and simple approach to evaluating models is to regress predicted values against observed values (or vice versa) and compare the slope and intercept parameters against the 1:1 line. Using the plot () function and xlab and ylab arguments, the values predicted by the fitted model were plotted against the actual values for both training and testing groups. The predicted values from the model were displayed on the x-axis and the actual values from the data set were displayed on the y-axis.

### 2.6 Comparison of developed models with each other

The performance of the models was measured using the predict () function by the R^2^ statistic for two groups of training and testing. Also, the rmse_vec () function was used to calculate the RMSE statistic for the predicted values. Using the obtained values, the efficiency of three models in predicting the response variables was measured. The higher the R^2^ and the lower the RMSE, the better the model will perform.

**Fig. 2:**
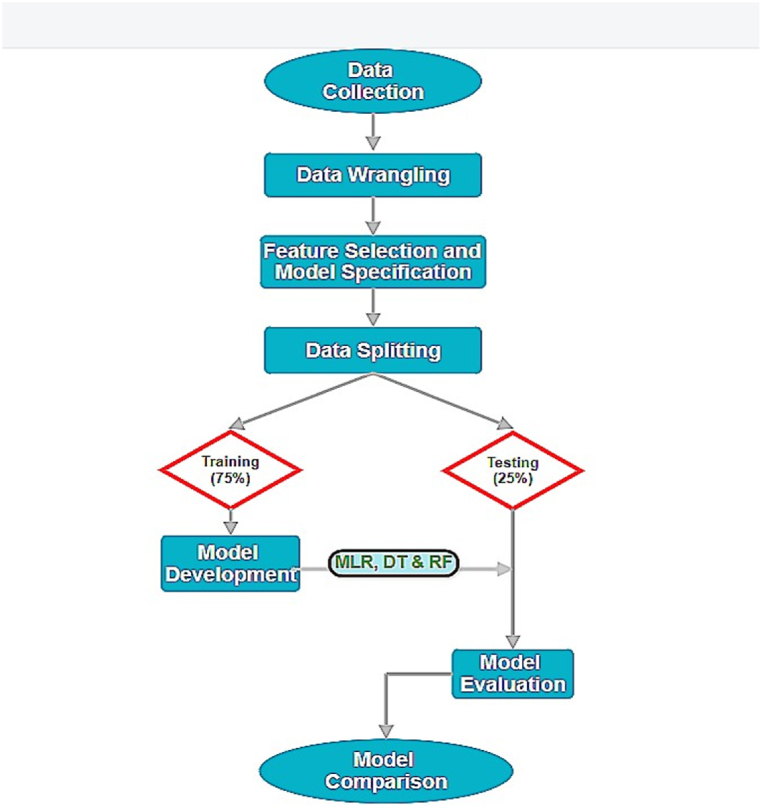
Sequential steps in the modeling process used in the study.

All the steps employed for modeling in this study are illustrated in Fig. 2.

## 3 Results and discussion

By splitting the data of each city into training and testing groups, for each response parameter the same training and testing groups were used to model with three mentioned approaches.

### 3.1 The correlation matrix

Based on the correlation coefficients matrix of quality parameters for groundwater resources in both cities shown in Fig. S1, the independent and dependent variables for predictive models of TH, TDS, and TA parameters based on other quality parameters were determined as follows.

• Groundwater resources of Kashan

TH ∼ Ca + Mg + SO_4_+NH_3_+F + Cl + HCO_3_+K + pH

TDS ∼ SO_4_+NH_3_+Na + HCO_3_+Cl + TH

TA ∼ HCO_3_+K + Na + pH + EC + Cl + SO_4_

• Groundwater resources of Aran Va Bidgol

TH ∼ SO_4_+Ca + Mg + TA + pH + K

TDS ∼ SO_4_+Na + Cl + Ca + NH_3_

TA ∼ HCO_3_+K + NH_3_+pH + Cl + TH

### 3.2 Performance of developed models

**Fig. 3:**
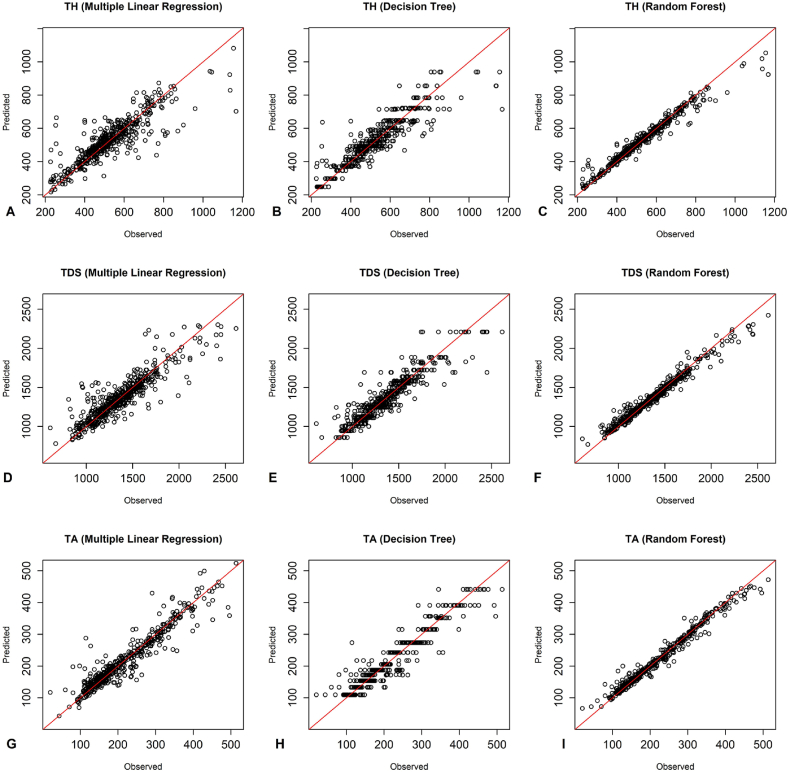
The performance of predictive models of the training group of Kashan groundwater resources - A-C) TH predictions using MLR, DT, RF. D-F) TDS predictions using MLR, DT, RF. G-I) TA predictions using MLR, DT, RF.

**Fig. 4:**
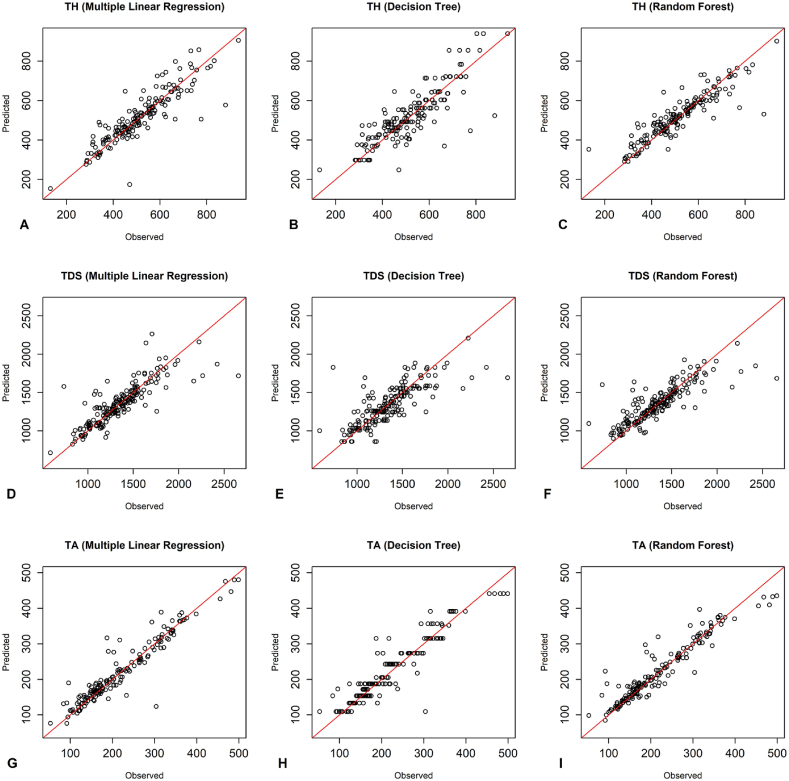
The performance of predictive models of the testing group of Kashan groundwater resources - A-C) TH predictions using MLR, DT, RF. D-F) TDS predictions using MLR, DT, RF. G-I) TA predictions using MLR, DT, RF.

**Fig. 5:**
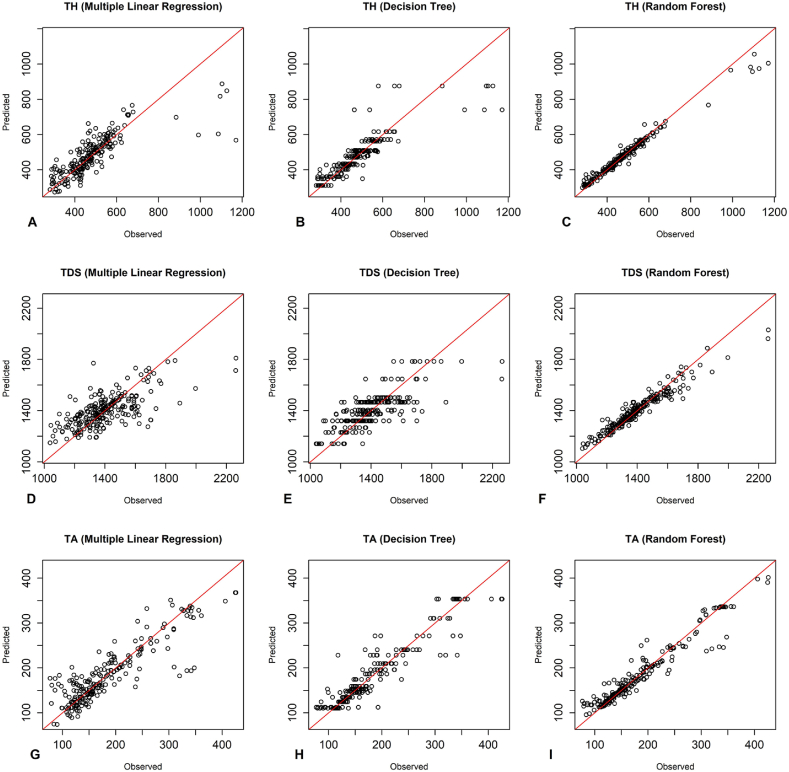
The performance of predictive models of the training group of Aran Va Bidgol groundwater resources - A-C) TH predictions using MLR, DT, RF. D-F) TDS predictions using MLR, DT, RF. G-I) TA predictions using MLR, DT, RF.

**Fig. 6:**
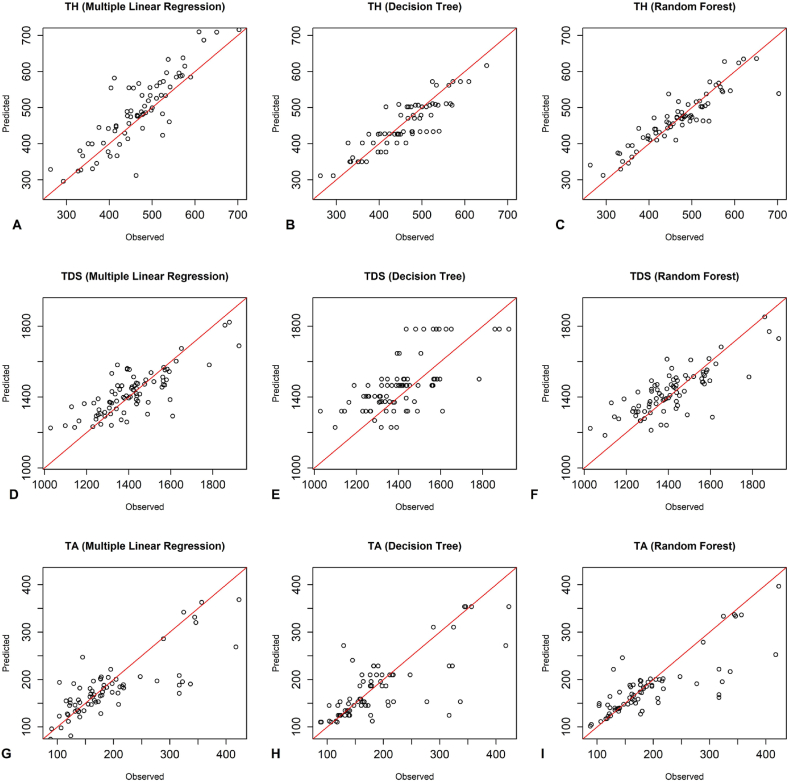
The performance of predictive models of the testing group of Aran Va Bidgol groundwater resources - A-C) TH predictions using MLR, DT, RF. D-F) TDS predictions using MLR, DT, RF. G-I) TA predictions using MLR, DT, RF.

According to Fig. 3, the observed and predicted values of the training group with the random forest model around the 1:1 line were more appropriate than the other two models for all three dependent parameters. As shown in Fig. 4, the observed and predicted values of the testing group with the RF model and MLR around the 1:1 line were more suitable than the DT model for all three dependent parameters. Additionally, Fig. 5 illustrates that the RF model was more appropriate than the DT model for all three dependent parameters, as the observed and predicted values of the training group aligned more closely with the 1:1 line. The plots of the observed and predicted values of the testing group show that the RF and MLR models around the 1:1 line was more matchable than the DT model in predicting TDS and TA parameters. Additionally, the MLR model demonstrated a better fit for the TH parameter (Fig. 6).

### 3.3 Measuring accuracy of models

As it is clear in Fig. S2 and Fig. S3 the coefficient of determination values obtained for all three developed models demonstrated the superior performance of the RF algorithm in predicting the response variables. Also, the coefficient of determination in the MLR model in predicting the response variables is notable. It is worth noting that the MLR model with a high determination coefficient of 0.7 performed better than the RF and DT models in predicting the TH values in the testing group of Aran Va Bidgol ground water resources.

In Fig. S4, it is evident that the RF model had lower RMSE values compared to the other two models when predicting the response parameter values of the training and testing groups in comparison to the actual values. According to Fig. S5, the RF model showed lower RMSE values compared to the other two models in predicting the response parameters' values of both the training and testing groups, in comparison to the actual data values.

### 3.4 Comparison of developed models

According to Table .1, the RF model had high performance with R^2^_=_ 0.96 and RMSE = 31.85 (mg/L CaCO_3_) in predicting the TH parameter of training group for Kashan groundwater resources. Also, it predicted the TH parameter of testing group well (R^2^_=_ 0.8 and RMSE = 59.32 (mg/L CaCO_3_)). The RF model showed high performance in estimating the TH parameter of the training group in Aran Va Bidgol groundwater resources (R^2^_=_ 0.97 and RMSE = 27.93 (mg/L CaCO_3_)). It is worth mentioning that the MLR model with R^2^_=_ 0.73 and RMSE = 56.71 (mg/L CaCO_3_) performed well in estimating the TH parameter of the testing group in Aran Va Bidgol groundwater resources.

**Table 1:** Comparison and overview of diverse Model Performances in Water Quality Studies.

Study Reference	Model Type	Parameter	Data	R^2^	RMSE
This study (Kashan)	MLR	TH	Training	0.7449	75.48 (mg/L CaCO_3_)
			Testing	0.7809	63.96 (mg/L CaCO3)
	DT		Training	0.8077	65.54 (mg/L CaCO3)
			Testing	0.7001	76.85 (mg/L CaCO3)
	RF		Training	0.9643	31.85 (mg/L CaCO3)
			Testing	0.8073	59.32 (mg/L CaCO3)
	MLR	TDS	Training	0.8043	134.19 (mg/L)
			Testing	0.6985	170.5 (mg/L)
	DT		Training	0.8550	115.51 (mg/L)
			Testing	0.6005	196.89 (mg/L)
	RF		Training	0.9713	57.24 (mg/L)
			Testing	0.6874	174.14 (mg/L)
	MLR	TA	Training	0.8998	28.8 (mg/L CaCO_3_)
			Testing	0.9027	28.27 (mg/L CaCO_3_)
	DT		Training	0.9070	27.74 (mg/L CaCO_3_)
			Testing	0.8814	31.51 (mg/L CaCO_3_)
	RF		Training	0.9791	13.65 (mg/L CaCO_3_)
			Testing	0.9021	28.44 (mg/L CaCO_3_)

This study (Aran Va Bidgol)	MLR	TH	Training	0.6252	85.5 (mg/L CaCO_3_)
			Testing	0.7394	56.71 (mg/L CaCO_3_)
	DT		Training	0.7106	75.13 (mg/L CaCO_3_)
			Testing	0.5199	81.09 (mg/L CaCO_3_)
	RF		Training	0.9750	27.93 (mg/L CaCO_3_)
			Testing	0.5527	59.69 (mg/L CaCO_3_)
	MLR	TDS	Training	0.4635	132.04 (mg/L)
			Testing	0.6120	102.85 (mg/L)
	DT		Training	0.5571	119.98 (mg/L)
			Testing	0.4324	141.4 (mg/L)
	RF		Training	0.9296	56.12 (mg/L)
			Testing	0.6144	103.25 (mg/L)
	MLR	TA	Training	0.7701	35.5 (mg/L CaCO_3_)
			Testing	0.6277	48.89 (mg/L CaCO_3_)
	DT		Training	0.8759	26.08 (mg/L CaCO3)
			Testing	0.5466	53.64 (mg/L CaCO_3_)
	RF		Training	0.9302	20.06 (mg/L CaCO_3_)
			Testing	0.6396	48.23 (mg/L CaCO_3_)
Farooq et al. [14]	MLR	TDS	–	0.73–0.95	4.5–11.3 %
			–	0.98	–
Aduojo et al. [28]	MLR	TDS	–	0.797	–
Abbas et al. [26]	MLR	TDS	Calibration	0.94	184.58 (ppm)
Al-Mukhtar and Al-Yaseen [20]	MLR	TDS	Validation	0.98	196.89 (ppm)
Nuraki et al. [22]	MLR	TDS	–	0.95–0.98	70.5–151.04 (mg/L)
		TH	–	0.99	1.28–1.9 (mg/L CaCO_3_)
Calvert [27]	MLR	TH	–	0.68233	86.61 (mg/L CaCO_3_)
		TDS	–	0.22112	151.60 (mg/L)
El Bilali and Taleb [25]	MLR	TDS	–	0.98	162.92 (mg/L)
	DT		–	0.75	764.14 (mg/L)
	RF		–	0.94	564.78 (mg/L)
Norki et al. [22]	RF	TDS	–	0.96–0.98	69.52–135.66 (mg/L)
		TH	–	0.95–0.99	18.69–51.56 (mg/L CaCO_3_)
Jena et al. [29]	RF	TDS	–	0.908	2.313 (mg/L)
Martinsen and Sand-Jensen [23]	RF	TA	–	0.66	0.132 (meq/L)
Towler et al. [24]	MLR	TA	–	0.95	11.8 (mg/L CaCO_3)_
Banerjee et al. [30]	DT	TA	–	1	–

	MLR	TA	–	0.0876	–
	DT	TH	–	0.1	–
	MLR	TH	–	0.0979	–
Pan et al. [31]	MLR	TH	–	0.995	–
Trabelsi and Bel Hadj Ali [32]	RF	TDS	Training	0.68	50 (mg/L)
		TDS	Testing	0.75	50 (mg/L)
M. Taşan, S. Taşan, Y. Demir [33]	RF	TDS	Training	0.952	96.572 (mg/L)
		TDS	Testing	0.861	194.234 (mg/L)
Adjovu GE, Stephen H, Ahmad S [34]	RF	TDS	–	0.8	–

Also, Table 1 shows that the RF model performed well in predicting the TDS parameter for Kashan groundwater resources in the training group, with R^2^ = 0.97 and RMSE = 57.23 mg/L. In predicting the TDS parameter in the testing group, the RF and MLR models both performed well. The RF model demonstrated excellent performance in forecasting the TDS parameter for Aran Va Bidgol groundwater resources in the training group, with R^2^ = 0.92 and RMSE = 56.12 mg/L. In the testing group, both the RF and MLR models performed well in predicting the TDS parameter.

The RF model also demonstrated high performance in predicting the TA in groundwater resources of Kashan, with R^2^ = 0.97 and RMSE = 13.64 mg/L. In the testing group of TA parameter, both the RF and MLR models performed well. The R^2^ and RMSE for the RF model in predicting TA values in the training group of Aran Va Bidgol groundwater resources were 0.93 and 20.05 mg/L CaCO_3_, respectively. In the testing group, values of 0.63 and 48.23 mg/L CaCO_3_ were obtained, indicating a high coefficient for the RF comparing the others (Table .1).

The Random Forest (RF) model demonstrates consistent excellence in various studies, underscoring its robustness and dependability for water quality analysis. Notably, the Kashan study's lower RMSE values, in contrast to those from Aran Va Bidgol, hint at the potential impact of regional factors or data characteristics on the model's predictive precision.

It is crucial to recognize that R^2^ measures the degree to which the model accounts for the data's variability, while the RMSE provides insight into the average magnitude of the prediction errors. Consequently, a model characterized by high R^2^ and low RMSE is generally considered both precise and trustworthy.

In a comprehensive analysis of various regression models applied to predict water quality parameters, the RF model has demonstrated exceptional accuracy and reliability. Specifically, the RF model's ability to predict TDS in the stations under study was reflected in high R^2^ values ranging from 0.96 to 0.98, indicating a strong correlation between observed and predicted values. The RMSE values, which measure the model's prediction error, were reported between 69.52 and 135.66 mg/L, suggesting a high level of precision in the model's predictions.

Similarly, the RF model's performance in predicting TH was also noteworthy, with R^2^ values between 0.95 and 0.99 and RMSE values ranging from 18.69 to 51.56 mg/L CaCO₃. These metrics underscore the model's robustness in accurately forecasting TH levels across the studied stations.

In contrast, the MLR model, while also showing strong predictive capabilities, exhibited slightly different performance metrics. For TDS prediction, the MLR model's R^2^ values were between 0.95 and 0.98, with RMSE values spanning 70.5–151.04 mg/L. For TH prediction, the MLR model achieved an R^2^ value of 0.99, with an impressively low RMSE range of 1.28–1.9 mg/L CaCO₃. These findings align with the results of the current study, indicating a consistent performance of the MLR model across different research contexts [22]. which is consistent with the results of the present study.

The study conducted by Martinsen and Sand-Jensen further corroborates these findings, where the RF model's efficacy in predicting TA was reported with an R^2^ value of 0.66 and an RMSE of 0.132 meq/L. This level of performance is consistent with the outcomes observed in the testing groups of the Aran Va Bidgol groundwater resource datasets, reinforcing the model's applicability in diverse settings [23].

Moreover, Towler et al.'s research demonstrated the MLR model's adeptness in predicting TA, with an R^2^ of 0.95 and an RMSE of 11.8, further validating the model's effectiveness as observed in the present study [24].

El Bilali and Taleb's investigation into agricultural water quality parameters in semi-arid regions using machine learning models revealed R^2^ values for TDS prediction at 0.98 for MLR, 0.75 for DT, and 0.94 for RF. The corresponding RMSE values were 162.92 mg/L for MLR, 764.14 mg/L for DT, and 564.78 mg/L for RF, with the RF model's R^2^ values affirming the findings of the current study [25]. Another research highlighted an R^2^ value of 0.797 for the MLR model in TDS prediction, which is in harmony with the present study's results [26]. The same study reported R^2^ and RMSE values for TH prediction using the MLR model as 0.68233 and 86.6078 mg/L CaCO₃, respectively, and for TDS prediction as 0.22112 and 151.6029 mg/L, respectively [27]. These values for the TH parameter are consistent with the findings of this study, confirming the anticipated outcomes.Overall, the RF model has proven to be highly effective in predicting TH, TDS, and TA parameters, outperforming other models in terms of accuracy and reliability. The MLR model also showed commendable performance, particularly in predicting total hardness in Aran Va Bidgol groundwater resources. The DT model, while effective, displayed lower performance compared to the RF model in predicting quality parameters. The R^2^ and RMSE values of the DT model in predicting the TA parameter for the training group of Kashan groundwater resources were 0.9 and 27.73 mg/L CaCO₃, respectively, and for the testing group, 0.88 and 31.51 mg/L CaCO₃, respectively. The DT model also showed good performance in predicting the TA values of Aran Va Bidgol groundwater resources in the training group, with an R^2^ of 0.87 and an RMSE of 26.08. These results highlight the potential of machine learning models in providing accurate predictions of water quality parameters, which is crucial for effective water resource management.

The study presents a significant advancement in the predictive modeling of water quality parameters, emphasizing the RF model's superior accuracy over MLR and DT models. This methodology proves especially beneficial for regions where traditional water quality testing poses logistical and financial challenges. The research enriches the existing body of knowledge by offering a comparative analysis of these predictive models, a facet not extensively covered in prior studies. By employing consistent training and testing groups for each response parameter across all models, the study ensures the reliability of its comparative performance analysis.

In terms of sustainable development, the study's approach aligns with global goals by providing a cost-effective and efficient strategy for water quality management, crucial for public health, ecosystem sustainability, and economic growth. The enhanced prediction accuracy bolsters informed decision-making, potentially leading to improved resource management and conservation efforts.

However, the study acknowledges limitations, including the potential lack of generalizability due to the specificity of the geographic data used. Additionally, the performance of the models may vary with different datasets or water quality parameters not considered in this study, indicating the need for broader validation. Despite these limitations, the study offers valuable insights into the application of machine learning in water quality assessment, contributing to the sustainability and resilience of water resource management practices.

## 4 Conclusion

The significance of water quality extends far beyond mere consumption; it is intrinsically linked to a myriad of critical aspects that form the bedrock of a thriving society. These aspects include, but are not limited to, human health, poverty alleviation, gender equality, food security, sustainable livelihoods, and the preservation of delicate ecosystems. Furthermore, water quality plays a pivotal role in fostering economic growth and facilitating social development. As such, safeguarding both the quality and quantity of water resources is not merely a technical challenge but a fundamental necessity for the sustainable management of these precious resources.

However, the traditional approach of sampling and analyzing groundwater resources to assess their quality presents significant logistical and financial challenges. It is a process that is often characterized by its high costs and labor-intensive nature, making it a less than ideal solution for regular monitoring. In light of these constraints, the application of statistical methods emerges as a vital tool. These methods serve as a safeguard, ensuring that the water provided to consumers meets the highest standards of safety and health.

In the realm of water quality assessment, predictive modeling has emerged as a revolutionary and cost-effective alternative to traditional methods. Predictive models offer a myriad of advantages: they are time-efficient, cost-saving, and user-friendly, granting easier access to crucial quality parameters of groundwater. This research delves into the efficacy of three distinct predictive models, developed using the R programming language, aimed at forecasting the quality parameters of groundwater resources in the arid central regions of Iran.

A comparative analysis of the performance of Multiple Linear Regression (MLR), Decision Tree (DT), and Random Forest (RF) models was conducted. The findings revealed a clear superiority of the RF model in developing robust predictive models for various water quality parameters. The MLR model also demonstrated commendable performance, particularly in predicting Total Hardness (TH) in the groundwater resources of Aran Va Bidgol. While the DT model was effective, it fell short of the RF model's predictive capabilities.

The implications of this study are profound, highlighting the potential to enhance the efficiency and cost-effectiveness of water quality monitoring strategies. Such advancements are instrumental in ensuring the provision of safe drinking water, a critical component of public health and well-being. The study's outcomes underscore the transformative impact that machine learning and predictive modeling can have on the management of water resources, paving the way for more informed and strategic decision-making in the realm of environmental sustainability.

Building upon the results and implications of the current study, future research prospects and suggestions could include.

1 Model Generalization: Testing the generalizability of the developed models across different geographical locations and water sources to validate their applicability on a broader scale.
2 Advanced Machine Learning Techniques: Investigating the application of more advanced machine learning techniques, particularly deep learning algorithms, to significantly enhance the precision and reliability of predictive models within the domain of water quality management. This exploration is aimed at leveraging the sophisticated capabilities of these advanced techniques to drive innovation and accuracy in environmental data analysis.
3 Water Policy Development: Utilizing the findings to inform and influence water policy, ensuring that regulations are based on accurate and up-to-date water quality data.
4 Community Engagement: Engaging local communities in the monitoring process, using citizen science approaches to gather more data and raise awareness about water quality issues.
5 Sustainability Assessments: Evaluating the sustainability of water resources by integrating predictive models with assessments of water usage, distribution, and conservation strategies.
6 Climate Change Effects: Investigating the effects of climate change on water quality and the predictive models' ability to adapt to changing environmental conditions.
7 Economic Evaluation: Conducting a cost-benefit assessments to determine the financial viability and impact of integrating predictive modeling techniques into water quality management systems, as opposed to relying on conventional methodologies. This analysis aims to provide a quantifiable measure of the economic implications associated with the adoption of such innovative approaches.

## Funding

The study was funded by the 10.13039/501100004484Tehran University of Medical Sciences [Grant No. 67704].

## Declarations

The authors of this article declare that they have no conflict of interests.

## Data availability statement

Data included in article/supp. material/referenced in article.

## Ethical approval

Not applicable.

## Consent to participate

Not applicable.

## Consent to publish

Not applicable.

## CRediT authorship contribution statement

**Aysan Morovvati Zarajabad:** Writing – review & editing, Writing – original draft, Visualization, Software, Methodology, Investigation, Data curation, Conceptualization. **Mahdi Hadi:** Writing – review & editing, Software, Methodology, Data curation, Conceptualization. **Ramin Nabizadeh Nodehi:** Writing – review & editing, Investigation. **Mahsa Moradi:** Writing – review & editing, Investigation. **Mohammad Rezvani Ghalhari:** Writing – review & editing, Investigation. **Abbas Zeraatkar:** Writing – review & editing. **Amir Hossein Mahvi:** Writing – review & editing, Supervision, Project administration, Investigation, Conceptualization.

## Declaration of competing interest

The authors declare that they have no known competing financial interests or personal relationships that could have appeared to influence the work reported in this paper.
