# Supplementary material for: Providing predictive models for quality parameters of groundwater resources in arid areas of central Iran: A case study of kashan plain
Source: Heliyon. 2024 May 17;10(11):e31493. doi: 10.1016/j.heliyon.2024.e31493 (PMC11152681; doi:10.1016/j.heliyon.2024.e31493)
Supplement: Multimedia component 4 [file mmc4.docx]

Supplementary Information

**Providing predictive models for quality parameters of groundwater resources in arid areas of central Iran: a case study of Kashan plain**

Aysan Morovvati Zarajabad ^a^, Mahdi Hadi ^b^, Ramin Nabizadeh Nodehi ^a^, Mahsa Moradi ^a^, Mohammad Rezvani Ghalhari ^a^, Abbas Zeraatkar^c^, Amir Hossein Mahvi ^a*^

**^a^**Department of Environmental Health Engineering, School of Public Health, Tehran University of Medical Sciences, Tehran, Iran

**^b^**Center for Water Quality Research (CWQR), Institute for Environmental Research (IER), Tehran University of Medical Sciences, Tehran, Iran

**^c^**Center for Monitoring Water and Wastewater Sanitation, Kashan Water and Wastewater Company, Kashan, Iran

**Content**

**Fig. S1** Correlation matrix of water quality parameters of (a) Kashan and (b) Aran Va Bidgol

**Fig. S2** Bar chart of the R^2^ values of the predictive models of the response parameters for the training and testing data sets of groundwater resources of Kashan

**Fig. S3** Bar chart of the R^2^ values of the predictive models of the response parameters for the training and testing data sets of groundwater resources of Aran Va Bidgol

**Fig. S4** Bar chart of the RMSE values of the predictive models of the response parameters for the training and testing data sets of groundwater resources of Kashan

**Fig. S5** Bar chart of the RMSE values of the predictive models of the response parameters for the training and testing data sets of groundwater resources of Aran Va Bidgol

|   a |
| --- |
|   b |

**Fig. S1** Correlation matrix of water quality parameters of (a) Kashan and (b) Aran Va Bidgol

**Fig. S2** Bar chart of the R^2^ values of the predictive models of the response parameters for the training and testing data sets of groundwater resources of Kashan

**Fig. S3** Bar chart of the R^2^ values of the predictive models of the response parameters for the training and testing data sets of groundwater resources of Aran Va Bidgol

**Fig. S4** Bar chart of the RMSE values of the predictive models of the response parameters for the training and testing data sets of groundwater resources of Kashan

**Fig. S5** Bar chart of the RMSE values of the predictive models of the response parameters for the training and testing data sets of groundwater resources of Aran Va Bidgol
